# Bibliographical Notice

**Published:** 1840-11-21

**Authors:** 


### MEDICAL EXAMINER.

DEVOTED TO MEDICINE, SURGERY, AND THE COLLATERAL SCIENCES.

No. 47.] PHILADELPHIA, SATURDAY, NOVEMBER 21, 1840. [Vol. III.

### BIBLIOGRAPHICAL NOTICE.

1. *On the Glanders in the Human Subject.* By John Elliotson, M. D. F. R. S., &c. (Medico-Chirurgical Transactions, London, 1830, Vol. xvi. p. 171—218.)
2. *Beiträg zu den Erfahrungen über die Schädliche Einwirkung des Rotzgifts auf Menschen.* Von Dr. W. Eck. *Observations on the Baneful Influence of the Poison of Glanders in Man.* By Dr. W. Eck. (Medicinische Zeitung for 3d and 9th May 1837, Nos. 18 and 19.)
3. *De la Morve et du Farcin chez l'Homme.* Par M. P. Rayer, Medecin de l'Hopital de La Charité, Medecin consultant du Roi, Membre de l'Academie Royale de Medicine. *On Glanders and Farcy in Man.* By M. P. Rayer, Physician to the Hospital of La Charité, &c. &c. (Memoires de l'Academie Royale de Medecine, Paris, 1837, Vol. vi. p. 625—872.) With two coloured Engravings.

These are the titles of Monographs published during the last ten years, upon a disease which, although evidently recognised since the year 1811, as is exhibited by some sparse observations, was not till the year 1830, the date of Dr. Elliotson's publication, erected into a distinct malady in the human subject and connected with its proximate cause, contagion from the horse or ass.

These works are fully reviewed in the Edinburg Med. and Surg. Journal for Oct. last, but the able reviewer has offered no opinion either in regard to the intimate nature of the disease, or the indications for its treatment, and it is on this account that we are unwilling to permit the works to pass unnoticed. Since their appearance, a continued succession of detailed cases, copied into our columns from the European periodicals, exhibiting the frequency and fatality of the affection in man, render us anxious to call the attention of our Southern brethren more particularly to the subject.

The reviewer states that "from the earliest records of the veterinary art the horse has been described as subject to two diseases of a fatal character, glanders, and farcy." Each of these have been divided into acute and chronic, making four forms, which, however, subsequent examinations have shown not to be distinct, but mere modifications, the one of the other. Thus, in animals, the inoculation of the matter from glanders, whether taken directly from a diseased horse, or from a man secondarily infected, will sometimes produce glanders, and sometimes farcy, and, "vice versa," the matter from farcy is equally capable of developing either affection. This mutual convertibility is not explained by the authors or their reviewers, and hence, contrary to our usual plan, we venture upon an explanatory suggestion, without a sufficient number of facts to prove the correctness of our view, in order to attract attention to the subject and solicit those facts upon which alone a permanent explanation can be based.

Is it not probable that in both diseases the nature of the poison is the same, and that the varieties depend only on the direction of this poison upon different humours? We assume the identity of cause from the results of inoculation. Let us study the difference in symptoms between glanders and farcy in animals, and judge from them whether our ground is tenable.

Each form of the affection is clearly of a typhoid nature, characterised by languor and debility at first, and afterwards by symptoms much less equivocal, which in their turn are reduced to certainty by post mortem examinations. It is true that in both, and particularly in glanders, an increased mucous secretion from the Schneiderian membrane is one of the first evidences of the existence of the disease, and it is a singular, and as yet inexplicable, fact that a pustular eruption in the nostrils is the most prominent and constant alteration of the solids exhibited in animals, and that it exists equally as a characteristic when communicated by contagion to man; the pustules themselves are peculiar in their form, umbilicated in the early stage like those of small pox, and subseqntly degenerating into ulcers, "strongly resembling syphilitic sores, having thickened margins and depressed centres," thus tempting us almost to ascribe to them a specific character, but these lesions of the solids existing alone would only constitute an infirmity, and could never be considered as the cause of death—for a knowledge of the intimate nature of the disease and its rapidly fatal termination we must recur to humoral pathology, and the difference between the two forms of the affection are explicable only on the same ground.

The sores enlarge. "It is at this stage that the discharge becomes bloody and offensive. Pustular eruptions break out on various parts of the body; the breathing becomes laborious, and is attended with a short thick cough; the submaxillary lymphatic glands become enlarged, and swollen lymphatic cords and glands may be seen and felt in various parts of the body. The *conjunctiva* and *membrana nictitans* become inflamed, and often assume a purple hue, and discharge a puriform humour. The debility of the animal increases, the legs become œdematous, and frequently, before death, sero-sanguinolent and sero-purulent abscesses have formed in these parts. The debility, tickling cough, emaciation, &c. rapidly increase; gangrenous and ecchymosed spots appear on various parts of the body; the hair becomes dry and harsh, and falls off on the slightest touch; and the animal dies in from nine to twenty-five days from the commencement of the symptoms.

The appearances presented on dissection are injection, reddening, thickening and ulceration of the nasal membrane; occasionally gangrenous spots here and there, and caries of the nasal bones and cartilages. The lining membranes of the larynx, trachea, and bronchii, are usually injected, thickened, and ulcerated, and covered with tough muco-purulent matter. *Ecchymosed spots are frequently noticed on the surface of the lungs; and numerous hard tubercles, about the size of a nut, are dispersed through their substance.** Sometimes these tubercles are hard, but friable, whitish towards their centre, but of a bright red towards their circumference; at other times they are reduced to the state of a muco-purulent deposit. The substance of the lung is very easily torn in the neighbourhood of these tubercles, and the whole lung has a swollen and injected appearance, is much softened in its texture, and when cut into often exhibits sero-purulent infiltration. The lymphatic glands, especially the submaxillary nasal, axillary, inguinal and bronchial, are much enlarged, indurated, and injected. Serous effusions are common in the cavities of the brain, chest, and abdomen, and collections of purulent or sero-purulent matter are commonly met with in various parts of the body. The spleen and liver are often found softened, whilst the kidneys are ulcerated, and filled with purulent matter.

Farcy, again, in the horse, generally appears in the form of small tumours on the legs, lips, face, neck, or other parts of the body, and hard cords may be seen and felt stretching between these. As the disease advances the tumours enlarge, become soft, burst, and degenerate into foul ulcers, with hard elevated edges, a depressed glassy centre, and an unhealthy purulent and bloody discharge. The horse loses its appetite, its limbs become œdematous, lameness ensues, and some degree of hectic fever comes on. In general at this period the nose begins to discharge, the animal is attacked with difficult respiration and cough, and dies with all the symptoms of glanders. Dissection usually discloses the same morbid alterations of structure noticed above as occurring in the glandered horse."

From this description we are entitled to infer that no other differences exist between glanders and farcy, than such as occur from the influence of any other morbid agent introduced into the system, under circumstances enabling this agent chemically to react upon the fluids returning to the centre of circulation, and thereby effect their decomposition. These fluids are venous blood and lymph. In the first the effects are the immediate decomposition of the whole blood, from the morbid agent entering at once into the torrent of circulation. In the second mere decomposition of one of the elements of the blood, and an arrest, temporary or permanent, of the poison by the lymphatic ganglia which the altered fluid is forced to traverse before entering into the great circulatory system.

The symptoms bear us out in this view—in glanders—dissolved blood, as shown by petichiæ, &c. during life—death in from 9 to 25 days—and after death metastatic abscesses, which are absolutely pathognomonic of purulent decomposition of this fluid. While in farcy we find, first, superficial tumours with hard cords between them, apparently local and evidently confined to the lymphatic system, and only subsequently changes characteristic of general disorganization of the humours, at which period the farcy clearly merges into glanders, and the two affections become identical.

The relation between glanders and farcy appears, then, to be analogous to that between the phlebitis and angeioleucitis which may result from ordinary wounds. The diseases are probably identical in regard to the general morbid

* The italics are our own.

action, and only differ in this, that while the purulent alteration of the fluids exists in all; in the two former (glanders and farcy) they are connected with an unknown element, apparently specific in its character, from which they derive their origin, and which continues to complicate them during the whole course.

The number of cases collected in the above works, and those published in late periodicals, exhibit then the fact, that in man glanders is of very frequent occurrence, far more frequent than hydrophobia, and is invariably fatal—that the symptoms are analogous to those already described in animals, and that it can be traced in the great majority of instances to direct contagion, while in others from the absence of the specific sore of inoculation, it has been supposed to originate in infection. In either case, although the local symptoms and the period of incubation may vary, the progress of the disease is identical and the termination the same; death, in two-thirds of the cases, before the 17th day.

Now, in purulent phlebitis, resulting from wounds, the same rigors usher in the disease, the same lassitude and cerebral symptoms are among the most prominent signs. The same typhoid state characterises it throughout—the same result invariably terminates it—and the same alterations are found after death, with the exception of those of the nasal mucous membrane, which may fairly be referred to the specific nature of the cause. We are unable to compare the average duration of the diseases, for in glanders we find simply *that death occurs before the seventeenth day*, while in phlebitis from wounds death frequently occurs at a much earlier period after the first rigor. Thus out of 12 personal observations of the latter disease, 2 died on the second day, 2 on the fourth, 3 on the fifth, 2 on the sixth, 1 on the ninth, and 1 on the thirty-first day. The general average duration of phlebitis is, however, we believe, from 5 to 9 days.

We refer to this subject cursorily at present, intending in our future numbers to dwell more at length on the importance of humoral pathology, but wishing merely for the moment to attract the attention of those of our brethren who are placed in circumstances favourable to the observation of these diseases in the horse aud in man, to the importance of their study, and to ask from them observations which will throw light upon their pathology and treatment.

